# Blood Cholesterol Trends 2001–2011 in the United States: Analysis of 105 Million Patient Records

**DOI:** 10.1371/journal.pone.0063416

**Published:** 2013-05-10

**Authors:** Harvey W. Kaufman, Amy J. Blatt, Xiaohua Huang, Mouneer A. Odeh, H. Robert Superko

**Affiliations:** 1 Quest Diagnostics, Madison, New Jersey, United States of America; 2 Quest Diagnostics, West Norriton, Pennsylvania, United States of America; 3 Celera (a Quest Diagnostics company), Alameda, California, United States of America; 4 Saint Joseph’s Hospital of Atlanta, Atlanta, Georgia, United States of America; Tulane School of Public Health and Tropical Medicine, United States of America

## Abstract

**Objectives:**

We report annual trends in low density lipoprotein cholesterol (LDL-C) from an in-care patient population of nearly 105 million adults across the United States (U.S.), from 2001 through 2011.

**Background:**

Average blood cholesterol values have declined in the U.S. since at least 1960. The National Health and Nutrition Examination Survey (NHANES) reported declining blood cholesterol values from 1999 through 2010. In the absence of more recent published data, we examined LDL-C values from a single clinical laboratory database to determine whether these values continued to decline through 2011.

**Methods and Results:**

We extracted almost 247 million LDL-C results from nearly 105 million adults who received diagnostic testing from a single national clinical laboratory. Annual age-adjusted mean LDL-C values were calculated, and analyzed by gender. Piecewise regression analysis of the total study population indicates a breakpoint, or change in slope, in the years following 2008 (F = 163.13; p<0.05). Between 2001 and 2008, the average rate of annual decline was −2.05 mg/dL (95% CI [−2.35, −1.75]). After 2008, mean LDL-C levels flattened out, with a slope not statistically different from zero (slope = −0.10 mg/dL/year; 95% CI [−1.46, 1.26]). This stabilization was observed in both genders and all age ranges, and was also reflected in the percentage of results in low- and high-risk categories.

**Conclusions:**

The trends reported suggest historical progress in decreasing LDL-C levels, observed from 2001–2008, may have stalled in recent years. Further research is needed to determine the cause of the observed trends and develop new strategies to reduce lipid-based cardiovascular risk further.

## Introduction

Average blood cholesterol values, the primary cardiovascular disease biomarker, have declined in the United States (U. S.) since at least 1960 [1], [2], [3], [4]. Between 1999 and 2010, the National Health and Nutrition Examination Survey (NHANES) reported blood cholesterol values were decreasing. The decline in hypercholesterolemia, observed in the past decade, is most likely associated with a number of physiological and behavioral factors, such as an increased awareness of lipid disorders, increased use of lipid-lowering medication (i.e., statins), improvements in diet, and reductions in trans-fat consumption [5], [6].

These trends are also reflected in the mortality rates attributable to cardiovascular disease, which declined by approximately 60% from 1970 through 2000, and by 30.6% from 1998 through 2007. Approximately 47% of the decline in coronary heart disease from 1980 to 2000 in the U.S. was attributable to increased use of evidence-based medical therapies, while 44% was attributable to changes in risk factors associated with lifestyle and the environment [7]. Despite these improvements, cardiovascular disease still accounted for one in three deaths in the U.S. in 2007 [8].

Building on this research, the American Heart Association (AHA) 2020 Strategic Impact Goals target a 20% relative improvement in overall cardiovascular health for all Americans, using a combination of four health behaviors (smoking, diet, physical activity, and body weight) and three health factors (glucose, cholesterol, and blood pressure) metrics [9].

The purpose of this study is to provide more recent trends in low density lipoprotein cholesterol (LDL-C) across a wide spectrum of patients representative of real-world medical practices across the U. S. We examined almost 247 million results derived from a single clinical laboratory database from an in-care patient population of nearly 105 million adults, and analyzed these 11-year trends through 2011 by gender and age ranges.

## Methods

Quest Diagnostics has over 145 million patient encounters each year across the United States. Test results are stored in the Quest Diagnostics Informatics Data Warehouse, which is the largest private clinical laboratory data warehouse in the United States and stores approximately 3 billion test results annually. For this Quest Diagnostics Health Trends® study, we extracted testing data for individual patients as described below; all data were de-identified prior to analysis. This study was determined to be exempt from institutional review by the Western Institutional Review Board.

The LDL-C testing methodology was consistent throughout the study period of January 1, 2001 through December 31, 2011 (total cholesterol and triglyceride reagents were from Beckman Coulter, Inc., Brea, California; the HDL-C reagent was from Roche Diagnostics, Indianapolis, IN). All testing was performed with Olympus analyzers (Beckman Coulter). LDL-C was calculated only if the triglycerides concentration was ≤400 mg/dL [10]. Accordingly, specimens were not included in this analysis if the triglycerides concentration exceeded 400 mg/dL. For the measured components of the lipid panel, the coefficient of variation of the control materials during the study period was consistently <5%.

Patients were included in the study if they were at least 18 years old at the time of the blood collection event and the ordering physician requested a lipid panel with a calculated LDL–C test during our 11-year study period. Patients ages 18 and 19 years were included to provide insights into this younger age cohort.

To measure long-term trends, we calculated annual age-adjusted mean LDL-C values from calendar years 2001–2011, using the Census 2000 population as the standard population [11], [12]. For patients with multiple test results during a calendar year, the annual mean LDL-C level was calculated and used in the analysis for that year; hence, every patient had only one value entered into the analysis dataset for each calendar year. Likewise, the average age of each patient was calculated during that year and was entered into the analysis dataset for that calendar year.

In addition, we also examined the percentage of the patient population that was in each of the following LDL-C categories each year (as defined by the National Cholesterol Education Program Expert Panel on Detection, Evaluation, and Treatment of High Blood Cholesterol in Adults, Adult Treatment Panel III [13]): <100 mg/dL, 100 to 129 mg/dL, 130 to 159 mg/dL, and ≥160 mg/dL.

Piecewise regression analysis was used to assess the existence and placement of breakpoints, as well as the non-linearity of the trends between 2001 and 2011 (a logit transformation was used when the data are presented as percentages); analysis of covariance was performed to assess differences in trends between genders and age groups; an analysis of variance was performed to assess the differences among age groups, in each year. The Bonferroni method was used in the analysis of variance to adjust for multiple comparisons [14]. Levels of statistical significance were set at p<0.05 for all tests. SAS 9.2 (SAS Institute Inc., Cary, North Carolina) was used for all data analyses.

## Results

### Age and Gender Distributions of Study Population in 2001 and 2011

The age and gender distributions of the study population remained relatively stable throughout the study period. The proportion of women increased slightly (from 54.3% in 2001 to 55.0% in 2011; Table 1). Overall, mean age increased by approximately 3 years (from 54.8 in 2001 to 57.8 years in 2011).

**Table 1:** Age and gender distribution of study population at endpoints of study period (2001 and 2011).

Age Group	2001 Total	2001 Males	2001 Females	2011 Total	2011 Males	2011 Females
(Years)	Number (%)	Number (%)	Number (%)	Number (%)	Number (%)	Number (%)
18 to 19	92,552 (0.7)	40,727 (0.7)	51,825 (0.7)	200,328 (1.0)	88,906 (0.9)	111,422 (1.0)
20 to 29	840,456 (6.2)	346,164 (5.6)	494,292 (6.7)	1,306,974 (6.3)	542,814 (5.8)	764,160 (6.7)
30 to 39	1,838,570 (13.5)	851,791 (13.7)	986,779 (13.4)	2,218,068 (10.7)	971,290 (10.4)	1,246,778 (10.9)
40 to 49	2,933,040 (21.6)	1,387,940 (22.3)	1,545,100 (20.9)	3,818,752 (18.4)	1,775,389 (19.0)	2,043,363 (17.9)
50 to 59	3,255,415 (23.9)	1,554,209 (25.0)	1,701,206 (23.0)	4,980,048 (23.9)	2,331,366 (24.9)	2,648,682 (23.2)
60 to 69	2,312,363 (17.0)	1,089,741 (17.5)	1,222,622 (16.6)	4,360,664 (21.0)	2,002,505 (21.4)	2,358,159 (20.6)
70 to 79	1,653,456 (12.2)	699,323 (11.3)	954,133 (12.9)	2,544,588 (12.2)	1,130,205 (12.1)	1,414,383 (12.4)
80+	668,313 (4.9)	240,496 (3.9)	427,817 (5.8)	1,365,101 (6.6)	517,317 (5.5)	847,784 (7.4)
Total	13,594,165 (100)	6,210,391 (100)	7,383,774 (100)	20,794,523 (100)	9,359,792 (100)	11,434,731 (100)

### LDL-C Trends Over 11 Years

246,717,189 test results from 104,690,326 patients aged 18 years and above were included in the analysis. Because average LDL-C values and average ages were used for each patient during each year, there were 195,050,298 observations in the dataset.

**Figure 1:**
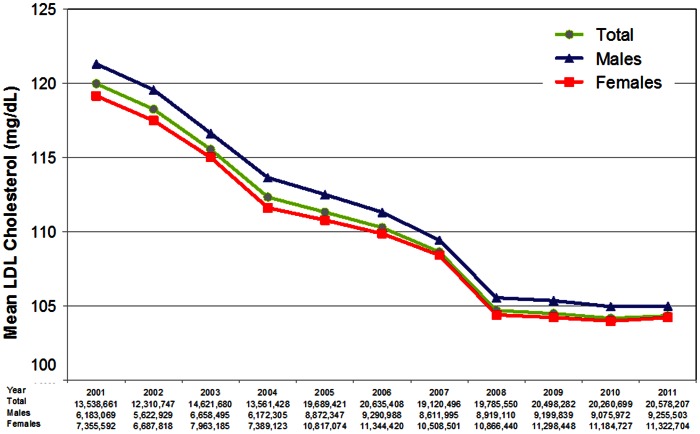
Annual age-adjusted mean LDL-C levels for the total population, and by gender, 2001–2011. 95% confidence intervals (not shown) range from ±0.1 mg/dL to ±0.3 mg/dL for all groups.

Over the 11-year period, there was a net 13% decline in the annual age-adjusted mean LDL-C level for the entire study population, from 120.0 mg/dL in 2001 to 104.3 mg/dL in 2011 (Figure 1). Piecewise regression analysis indicates a breakpoint, or change in slope, in the years following 2008 (F = 163.61; p<0.05; MSE = 0.67) (Table 2). Between 2001 and 2008, the average rate of annual decline was −2.05 mg/dL (95% CI [−2.35, −1.75]). The annual age-adjusted mean LDL-C declined from 120.0 mg/dL in 2001 to 104.7 mg/dL in 2008. After 2008, mean LDL-C levels flattened out, with a slope not statistically different from zero (slope = −0.10 mg/dL/year; 95% CI [−1.46, 1.26]). The annual age-adjusted mean LDL-C was 104.5 mg/dL in 2009 and 104.3 mg/dL in 2011.

**Table 2:** Piecewise regression results for the total study population, and by gender.

Parameter^a^	Overall Population Estimate [Approximate 95% CI]	Male Population Estimate [Approximate 95% CI]	Female Population Estimate [Approximate 95% CI]
Intercept of Segment 1 (2001 to 2008)	4221.8 [3625.3, 4818.4]	4367.0 [3819.2, 4914.9]	4047.7 [3413.3, 4682.2]
Slope of Segment 1 (2001 to 2008)	−2.05 [−2.35, −1.75]	−2.12 [−2.40, −1.85]	−1.96 [−2.28, −1.65]
Slope of Segment 2 (2009 to 2011)	−0.10 [−1.46, 1.26]	−0.18 [−1.44, 1.07]	0.001[−1.45, 1.45]
F-statistic	163.61^b^	209.37^b^	132.83^b^
MSE	0.67	0.56	0.75

a The piecewise regression equation for the period of study prior to the estimated breakpoint is: y = a1+b1*year. The piecewise regression equation for the period of study after the estimated breakpoint is: y = a1+c*(b1−b2) +b2*year. (a1 = intercept of segment 1; b1 = slope of segment 1; b2 = slope of segment 2; c = estimated breakpoint.).

b p<0.05.

There was a net 13.4% decline in the annual age-adjusted mean LDL-C level for the male population, from 121.3 mg/dL in 2001 to 105.0 mg/dL in 2011 (Figure 1). Piecewise regression analysis indicates a breakpoint in the years following 2008 (F = 209.37; p<0.05; MSE = 0.56) (Table 2). Between 2001 and 2008, the average rate of annual decline was −2.12 mg/dL (95% CI [−2.40, −1.85]). The annual age-adjusted mean LDL-C declined from 121.3 mg/dL in 2001 to 105.5 mg/dL in 2008. After 2008, mean LDL-C levels flattened out, with a slope not statistically different from zero (slope = −0.18 mg/dL/year; 95% CI [−1.44, 1.07]). The annual age-adjusted mean LDL-C was 105.3 mg/dL in 2009 and 105.0 mg/dL in 2011.

There was a net 12.5% decline in the annual age-adjusted mean LDL-C level for the female population, from 119.1 mg/dL in 2001 to 104.2 mg/dL in 2011 (Figure 1). Piecewise regression analysis indicates a breakpoint in the years following 2008 (F = 132.83; p<0.05; MSE = 0.75) (Table 2). Between 2001 and 2008, the average rate of annual decline was −1.96 mg/dL (95% CI [−2.28, −1.65]). The annual age-adjusted mean LDL-C declined from 119.1 mg/dL in 2001 to 104.4 mg/dL in 2008. After 2008, mean LDL-C levels flattened out, with a slope not statistically different from zero (slope = 0.001 mg/dL/year; 95% CI [−1.45, 1.45]). The annual age-adjusted mean LDL-C was 104.2 mg/dL in 2009 and 104.2 mg/dL in 2011. Between 2001 and 2008, the slope coefficients for males and females were not significantly different (p = 0.711) (Figure 1).

**Figure 2:**
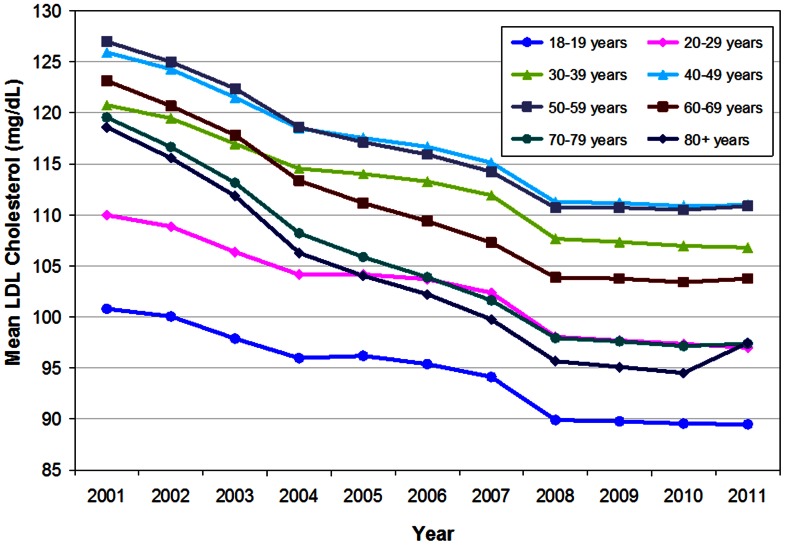
Annual mean LDL-C levels, by age group, 2001–2011. 95% confidence intervals (not shown) range from ±0.1 mg/dL to ±0.3 mg/dL for all groups.

Piecewise regression analysis of the annual mean LDL-C levels by age range indicates a breakpoint in the years following 2008 (F = 285.39; p<0.05; MSE = 0.87). Between 2001 and 2008, annual age-adjusted mean LDL-C levels declined significantly among all age ranges (p<0.05), and the rate of decline differed by age range (p<0.05) (Figure 2). Patients in the 80+ year age group had the greatest overall rate of decline (slope = −2.9 mg/dL/year), and patients in the 18–19 year age group had the smallest overall rate of decline (slope = −1.4 mg/dL/year)). Between 2009 and 2011, the rate of decline did not differ by age range (p = −0.374).

**Figure 3:**
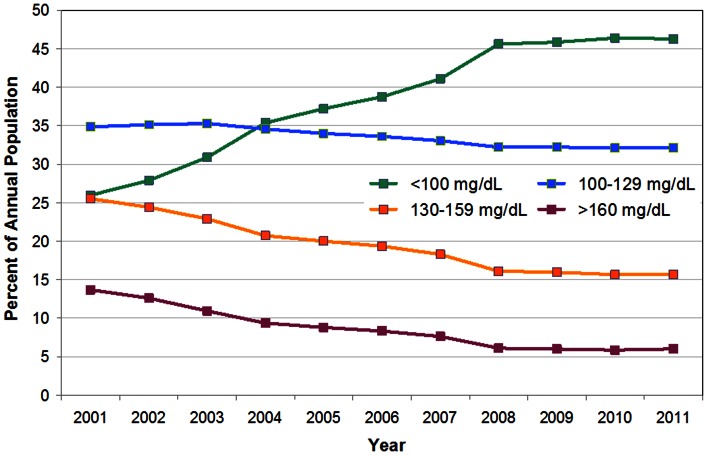
Distribution of LDL-C category, 2001–2011.

The percentage of patients with LDL-C of <100 mg/dL (classified as low-risk) increased from 26.0% in 2001 to 46.3% in 2011; while the percentage of patients with LDL-C of ≥160 mg/dL (classified as high-risk) declined from 13.7% in 2001 to 6.0% in 2011(Figure 3). Between 2001 and 2008, the percentage of patients with LDL-C of <100 mg/dL increased from 26.0% to 45.6%, while the percentage of patients with LDL-C of ≥160 mg/dL declined from 13.7% in 2001 to 6.1% in 2008. The percentage of patients with LDL-C of <100 mg/dL increased from 45.8% in 2009 to 46.3% in 2011, while the percentage of patients with LDL-C of ≥160 mg/dL was 5.99% in both 2009 and 2011.

Piecewise logit regression analysis of the percentage of patients in low- and high-risk categories indicates a breakpoint in the years following 2008 (F = 1525.99; p<0.05; MSE = 0.0012).

## Discussion

The current study adds to the body of scientific knowledge by showing that the declining LDL-C trends previously reported by NHANES may have halted since 2008 [1,2). We examined the LDL-C results of nearly 105 million unique patients from 2001 to 2011, in what is believed to be the largest nationally-based retrospective study of LDL-C trends reported to-date. Other studies that have examined population trends in LDL-C have been constrained by smaller populations, shorter study periods, and smaller geographical coverage [1], [2], [3], [4]. Furthermore, our study reported data annually whereas most recently published studies report results in time periods that cover multiple years – which mask the plateau observed in our study. Finally, our study is the first large-scale study to report data from 2009, 2010 and 2011 [3], [4], [5].

In the current study, the annual age-adjusted mean LDL-C level in 2001 was similar to that reported by NHANES for the same time period (120.0 mg/dL), although the two studies are based on different study methodologies. In addition, the percent of individuals with desirable LDL-C levels (<100 mg/dL) in the earlier NHANES III period (1988–2004) is similar to for the current study’s findings for 2001 (23% versus 26%) [2].

In contrast to the gradual decline reported in NHANES, we observed a 13% decline in LDL-C from 2001–2011 (120.0 mg/dL to 104.3 mg/dL). Since every 10 mg/dL decline in LDL-C is associated with an approximately 5% to 13% reduction in major vascular disease events and mortality, the 16 mg/dL decline we observed is approximately equivalent to a 8% to 21% decline in cardiovascular disease mortality, without consideration of changes over time in other risk factors [15].

A key observation from the current study is that the decrease in LDL-C levels through 2008 halted in 2009 and has remained virtually flat since. This plateau was observed for all ages and both genders. It is beyond the scope of the current study to demonstrate how specific changes in medical practice and patient behaviors have affected our study’s observations. However, this observation suggests that the AHA goal of a 20% improvement in cardiovascular health may be difficult to obtain by 2020 [9].

The decline in annual age-adjusted mean LDL-C was greater among men than women during the 11-year period. Though not statistically significant, this difference may reflect meaningful differences in the prescription rate and effectiveness of lipid-lowering interventions, including statins and lifestyles, between the genders. Other studies have observed different effects of statins on men compared to women; some have indicated a possibly greater cardiovascular risk reduction among men [16], [17], while others suggested that statins were as effective in women as in men, or even more so [18]–[24]. Furthermore, the observed differences in LDL-C levels may, in part, be due to differences in under-appreciation of heart disease risk in women [25], as the first women-specific guidelines were published by the AHA in 1999, and new algorithms for risk classification in women were not published until 2007 [26], [27].

The current study has several limitations. By definition, the population in this study represents in-care patients from all 50 states and the District of Columbia seeking physician-ordered laboratory testing and does not necessarily reflect the population profiles of the general adult U.S. population. In addition, we did not have access to clinical information including changes in lifestyle and medication. Furthermore, although changes in laboratory methodology could account for apparent trends in LDL-C, this is unlikely since the laboratory methods were standardized throughout the study period [28]. In addition, Quest Diagnostics laboratories are all CLIA-certified and participate in standardization and proficiency testing programs. As noted in the Methods, analytical precision was consistent throughout the study period for all of the performing laboratories.

Population-based blood cholesterol levels have been declining in the U.S. for a long time, probably since at least the 1960s, when data from the National Health and Examination Survey (predecessor to NHANES) first became available. This study of nearly 105 million in-care patients, from a major laboratory database, reports a decline in LDL-C levels during the 2001 to 2008 period (for both men and women of all adult age ranges), followed by a period of stabilization from 2008 to 2011. Although many of our observations require more research to fully understand the underlying dynamics, our study suggests that our half-century of progress in improving LDL-C levels may have suddenly stalled since 2008.
